# Clinically useful prediction of hospital admissions in an older population

**DOI:** 10.1186/s12877-020-1475-6

**Published:** 2020-03-06

**Authors:** Jan Marcusson, Magnus Nord, Huan-Ji Dong, Johan Lyth

**Affiliations:** 1 Acute Internal Medicine and Geriatrics, Department of Health, Medicine and Caring Sciences, Linköping University, Linköping, Sweden; 2 Family Medicine, Department of Health, Medicine and Caring Sciences, Linköping University, Linköping, Sweden; 3 Pain and Rehabilitation Centre, Department of Health, Medicine and Caring Sciences, Linköping University, Linköping, Sweden; 4 Research and Development Unit in Region Östergötland, Department of Health, Medicine and Caring Sciences, Linköping University, Linköping, Sweden

**Keywords:** Prediction, Hospitalization, Older persons

## Abstract

**Background:**

The healthcare for older adults is insufficient in many countries, not designed to meet their needs and is often described as disorganized and reactive. Prediction of older persons at risk of admission to hospital may be one important way for the future healthcare system to act proactively when meeting increasing needs for care. Therefore, we wanted to develop and test a clinically useful model for predicting hospital admissions of older persons based on routine healthcare data.

**Methods:**

We used the healthcare data on 40,728 persons, 75–109 years of age to predict hospital in-ward care in a prospective cohort. Multivariable logistic regression was used to identify significant factors predictive of unplanned hospital admission. Model fitting was accomplished using forward selection. The accuracy of the prediction model was expressed as area under the receiver operating characteristic (ROC) curve, AUC.

**Results:**

The prediction model consisting of 38 variables exhibited a good discriminative accuracy for unplanned hospital admissions over the following 12 months (AUC 0.69 [95% confidence interval, CI 0.68–0.70]) and was validated on external datasets. Clinically relevant proportions of predicted cases of 40 or 45% resulted in sensitivities of 62 and 66%, respectively. The corresponding positive predicted values (PPV) was 31 and 29%, respectively.

**Conclusion:**

A prediction model based on routine administrative healthcare data from older persons can be used to find patients at risk of admission to hospital. Identifying the risk population can enable proactive intervention for older patients with as-yet unknown needs for healthcare.

## Background

With an increase in the aging population worldwide, older age is generally associated with increased health-related needs and increased healthcare costs – but not by as much as previously expected [1]. Nevertheless, the association with both healthcare utilization and costs varies [2, 3] and in some high-income countries healthcare costs per person actually fall significantly after the age of 75 [4, 5]. Differences in provider systems, in the management of frail older people and in cultural norms, particularly near the time of death, may contribute to the fact that the association between age and healthcare costs is also strongly influenced by the healthcare system itself [1].

Even though the future challenges for the healthcare system due to an aging population might have been exaggerated, the present healthcare situation for the elderly population in many countries is insufficient and not designed according to their healthcare needs [6]. The healthcare of the aging population relates to morbidity, multi-morbidity and frailty [7]. But, at the same time, several reports indicate that a majority of the aged population is satisfied with their health (see [8]), manage life at home and consider themselves to be healthy [9, 10]. Only a minority of the aged population needs hospital care. In most cases, the healthcare system does not separate the heterogeneous old-age population, but rather organizes both hospital and primary care using a passive and reactive (acting when symptoms or problems occur) approach.

In order to detect elderly people with significant care needs (hospital care), there have been many attempts to define “frail” older people [11–13]. In this context, however, scales used for the prediction of persons in need of healthcare, some of which are frail, exhibit some major shortcomings. Firstly, “frailty” is not an easily defined medical condition for which there is a consensus on its operational definition [13–16]. Secondly, and from a clinical perspective more importantly, evaluation using clinical instruments requires trained staff for each individual evaluation and is not always easily applied within a broader clinical context where a primary geriatric perspective may not always be present (primary care, acute ward disciplines). A final limitation of the use of “frailty” scales in a wider clinical context is the fact that most elderly people (75% of 80+) seem to manage themselves at home, despite multi-morbidity and frailty. This was indicated in two separate studies on 85-year-olds (England, Sweden), concluding similar pictures of health and aging [9, 10]. A majority (> 75%) of the studied 85-year-olds managed their lives at home, rated themselves as healthy (80% rated their health good to excellent) and seldom used hospital care. Only $\frac{1}{4}-\frac{1}{3}$ of the aged population appeared to be high consumers of healthcare. These facts underline the difficulty of managing healthcare in an aged community. Our ability to detect individuals with possible needs, and to direct the care resources specifically towards those with greatest need of care prior to hospitalization, is not optimal.

Statistical or digital prediction models have been suggested as an evidence-based method to identify or select older persons in greater need of healthcare [17]. Earlier studies indicated that administrative data are useful in the prediction of hospital care [18], also for older adults in a group health cooperative [19]. More recently the use of a use of electronic administrative data to identify older community dwelling adults at high risk for hospitalization demonstrated good accuracy (AUC 0.678) [20]. In the present study we wanted to investigate a larger county population not limited to health insurance systems or other selection factors, to see whether we could develop a digital prediction model for older adults at high risk for hospital care that can be used in routine healthcare. If this group of elderly could be identified, proactive healthcare activities can be considered before hospital care takes place [21]. And some persons in need of hospital care could be directed to an appropriate clinic for care, instead of using the emergency care system.

## Methods

This prediction model study is reported in accordance with the TRIPOD checklist [22].

### Aim, design, setting and population

The aim was to develop and test a clinically useful model for predicting hospital admissions of older persons based on routine healthcare data. This is a prospective cohort study that included all residents aged 75–109 years in the county of Östergötland (*n* = 40,728) located in the south-east of Sweden. This age group constitutes 9.6% of the population, close to the national proportion of 9.2%. In the county of Östergötland, healthcare for the elderly is provided mainly by 43 healthcare centres in primary care and four hospitals, one of which is the University Hospital of Linköping.

### Data source and study variables

The 12-month data were obtained between November 2015 and October 2016 from the computerized information system of the County Council of Östergötland, where statistics for all healthcare in the county are stored. For example, for the whole population there are records of the number of visits to primary or hospital care, number of days in hospital, diagnostic codes for each visit etc. We used unplanned in-ward hospital stays between November 2016 and October 2017 as the dependent variable. Several time periods were tested and the predicted cases were included in a intervention study [21]. We included number of physician visits, number of non-physician visits (to nurses, occupational therapists or physiotherapists), number of previous in-ward hospital stays, number of emergency room (ER) visits, age, gender and International Classification of Diseases, and 10th Revision, (ICD10)-codes grouped by two digits. For each diagnosis, two variables were constructed, one based on open-clinic visits and one based on hospital visits. To get good precision in the estimation of the coefficients and to get a reliable model over time, variables with number of observations less than 40 were excluded. All diagnosis variables were dichotomized into yes or no. People who died during the following prediction period were included in the analysis.

### Model developing

The data was randomly divided into two halves, a training data set and a validation data set. The training set was used to build a prediction model and the validation set was used to validate this model. The prediction model algorithm was developed using multivariable logistic regression (LR) with forward selection) (see statistics below). The aim was to identify participants aged 75 or older who are likely to be hospitalized within the next 12 months.

### Statistical analysis and external validation

The first step was to calculate the univariable association for each variable with 12-months unplanned hospital admission. Because of large number of observations that could result in statistical significance for rather weak associations, only variables with *p*-values less than 0.001 was further included in the multivariable analysis.

Multivariable logistic regression was then used to identify significant factors predictive of unplanned hospital admission over a 12-month period. The model-building process consisted of three steps: selecting the variables, building the model, and validating the model. The best model was assessed by change in Akaike information criterion. A penalty factor of five was used to avoid overfitting and to reduce the number of variables in the final model. Collinearity was observed by calculating variance inflation factor for each variable in the final model and variables with a value above five were excluded. After the final model was made some further test was done in an attempt to further improve the model. First, we tested all 2-way interactions. Further, we tested to log-transform all numerical variables. Finally, we tested non-linearity for numerical variables by using restricted cubic splines. If an improvement in AUC was not achieved, the simplest model was chosen because we wanted a robust model that was easy to implement. Risk scores were calculated for all individuals.

Model performance measures: Overall discrimination was assessed using c-statistic, a measure of goodness of fit for binary outcomes in a logistic regression model. The area under the receiver operating characteristic (ROC) curve (AUC) is used to quantify the binary outcomes (hospital admission or not). The ROC curve is continually plotting every ideally possible sensitivity versus specificity across all threshold cut-off points. AUC reflects the accuracy of the predictive models and can be compared among the different models. AUC 0.5 means the model has no discrimination (the proportions of true cases and false positive cases are equal) whereas AUC 1.0 means the model has a perfect discrimination [23]. Five different sensitivity analyses were performed to assess how the prediction model changed in different settings. The first model included both unplanned and planned hospital admissions, the second model excluded people who died within the 12-month follow-up period and in the last two models, different follow-up periods 3-, and 6 months was tested. Lastly, we tested the least absolute shrinkage and selection operator (lasso) as an alternative selection method.

External validation was also performed in two additional data sets. One using the same time period as above but including ages 65–74 (*n* = 51,104). And another using the age group 75+ for year 2012 for prediction of unplanned hospital admission the following 12 months (*n* = 38,121).

All statistics were performed using R version 3.5.2 (R Core Team, Vienna, Austria). The Modern Applied Statistics with S (MASS) package was used for fitting the logistic model and the pROC package was used for estimating the AUC. The Lasso and Elastic-Net Regularized Generalized Linear Models (glmnet) package was used for fitting the lasso model. The Regression Modeling Strategies (rms) package was used for analysing with restricted cubic splines.

### Ethical aspects

The study has been subject to ethical evaluation and was approved by the regional ethical review board in Linköping (Dnr 2016/347–31).

## Results

In total, 40,728 individuals aged 75 years or older (57.7% women) were registered in the database. The demographic characteristics of these and their use of unplanned hospital care within 12-month subsequent period is given in Table 1. Even though the number of cases admitted to hospital (unplanned) decreased across the ages of 75 to 90+, the relative proportions of those in hospital increased (from 15 to 28%). Thus, it is more likely that a person 90+ years of age is admitted to hospital than a person aged 75–79.

**Table 1:** Characteristics of the population ≥ 75–109 years in relation to unplanned hospital admissions

Characteristic	Unplanned admission to hospital, n (%)		
	Train *n* = 20,364	Validation *n* = 20,364	Total *n* = 40,728
Total, *n* (%)	4130 (20.3)	4037 (19.8)	8167 (20.0)
Gender			
Male	1838 (9.0)	1834 (9.0)	3672 (9.0)
Female	2292 (11.3)	2203 (10.8)	4495 (11.0)
Age, years			
75–79	1328 (6.5)	1249 (6.1)	2577 (6.3)
80–84	1193 (5.9)	1119 (5.5)	2312 (5.7)
85–89	954 (4.7)	1014 (5.0)	1968 (4.8)
90+	655 (3.2)	655 (3.2)	1310 (3.2)

**Fig. 1:**
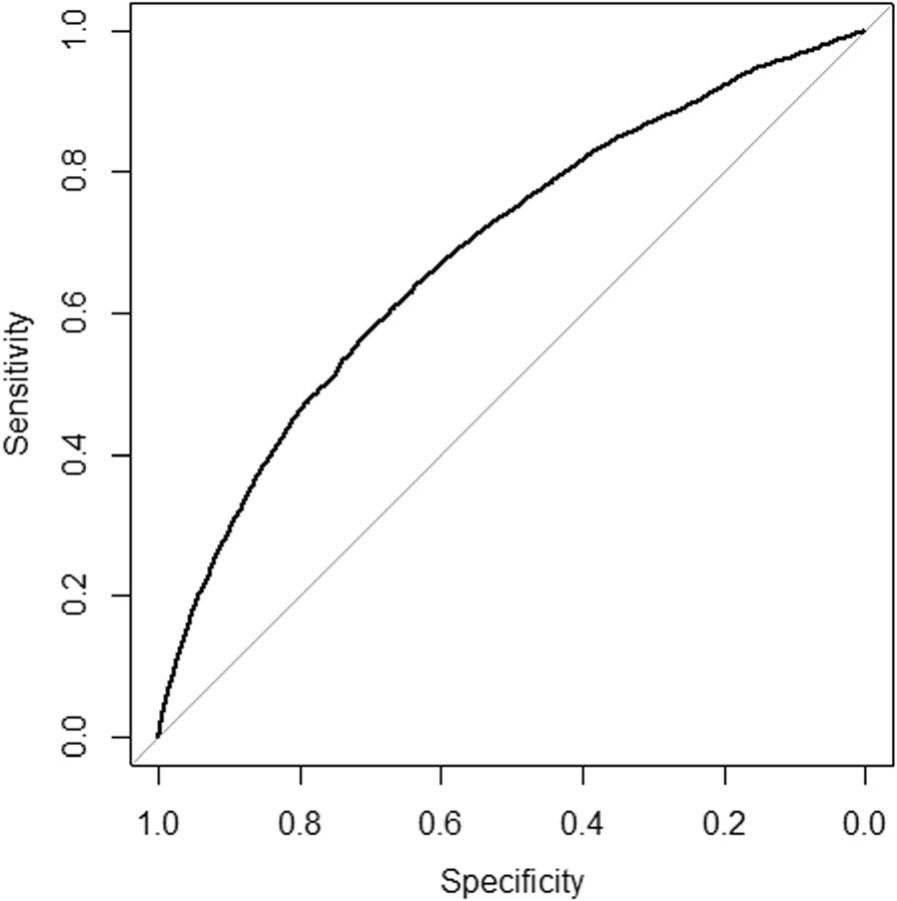
The ROC curve for predicting unplanned hospitalization derived from logistic regression using the validation data set (*n* = 20,364). Area under ROC curve (AUC) = 0.69, (95% CI 0.68–0.70)

In total, 650 variables were available for analysis where 233 showed a statistically significant (*p* < 0.001) association with 12-month unplanned hospital admission in the training data set. Table 2 presents the 20 most significant variables from the univariable analyses. The results from the multivariable final predictive model are presented in Table 3. The AUC of hospital admission over the subsequent 12 months was 0.69 (95% CI: 0.68–0.70) in the validation data set (Fig. 1). The best prediction variables were number of emergency-room visits, age, number of non-physician visits and number of physician visits, which alone resulted in an AUC of 0.67 (95% CI: 0.66–0.68)*.* No collinearity problem existed as the highest variance inflation factor was 2.1 for number of emergency room visits. We found statistically significant interactions between number of emergency room visits and number of physician visits, between number of emergency room visits and previous inpatient care and between number of emergency room visits and number of non-physician visits. However, the effects were very small and we could not improve the AUC in the final model. Neither could log-transformation of the numerical variables improve AUC. We found evidence of non-linearity for age and number of emergency room visits, but the non-linearity components were quite small and we could not improve the AUC. Because AUC was not improved, we decided to select the final model without further alterations.

**Table 2:** The twenty most significant variables predicting the risk for unplanned admission to hospital

		Number	% unplanned hospital admission	Crude OR	95% CI
Total		20,364	20.3	–	–
Categorical Variables					
Diagnoses in hospital care					
E11 Type 2 diabetes mellitus	No	19,718	19.5	1 (ref)	–
	Yes	646	43.3	3.15	(2.69–3.70)
I10 Essential hypertension	No	18,174	18.3	1 (ref)	–
	Yes	2190	36.5	2.57	(2.33–2.82)
I25 Chronic ischaemic heart disease	No	19,663	19.5	1 (ref)	–
	Yes	701	42.9	3.11	(2.67–3.63)
I48 Atrial fibrillation and flutter	No	19,235	19.0	1 (ref)	–
	Yes	1129	42.2	3.12	(2.76–3.53)
I50 Heart failure	No	19,712	19.4	1 (ref)	–
	Yes	652	47.5	3.77	(3.22–4.41)
J44 Chronic obstructive pulmonary disease	No	20,046	19.8	1 (ref)	–
	Yes	318	47.8	3.70	(2.96–4.62)
N18 Chronic renal failure	No	20,179	20.0	1 (ref)	–
	Yes	185	53.0	4.51	(3.37–6.04)
Z92 Personal history of medical treatment	No	19,596	19.3	1 (ref)	–
	Yes	768	44.9	3.41	(2.94–3.94)
Z95 Presence of cardiac and vascular implants and grafts	No	19,741	19.6	1 (ref)	–
	Yes	623	42.2	3.00	(2.55–3.53)
Diagnoses in open-clinic visits					
I25 Chronic ischaemic heart disease	No	18,294	19.2	1 (ref)	–
	Yes	2070	30.0	1.80	(1.63–1.99)
I48 Atrial fibrillation and flutter	No	17,808	18.5	1 (ref)	–
	Yes	2556	32.4	2.11	(1.92–2.31)
I50 Heart failure	No	18,936	18.9	1 (ref)	–
	Yes	1428	38.9	2.73	(2.44–3.06)
R06 Abnormalities of breathing	No	19,445	19.5	1 (ref)	–
	Yes	919	35.8	2.30	(1.99–2.64)
R07 Pain in throat and chest	No	19,504	19.6	1 (ref)	–
	Yes	860	36.3	2.34	(2.02–2.70)
Z51 Other medical care	No	19,431	19.5	1 (ref)	–
	Yes	933	37.3	2.46	(2.14–2.82)
Continuous Variables^a, b^					
Age		81	(75–106)	1.05	(1.04–1.05)
Emergency room (ER) visits		0	(0–25)	1.52	(1.47–1.57)
Non-physician visits		4	(0–210)	1.02	(1.02–1.03)
Physician visits		3	(0–100)	1.08	(1.07–1.09)
Previous in-ward hospital stays		0	(0–16)	1.56	(1.50–1.62)

^a^Medians were reported as appropriate for continuous variables. ^b^Range was reported as appropriate for continuous variables. *OR* Odds Ratio, *CI* Confidence interval. Hospital admissions within 12 months from the training sample (*n* = 20,364) expressed as crude odds ratios and 95% confidence intervals (CI) from univariable analysis. Variables are sorted by name and all *p*-values < 0.001

**Table 3:** The final predictive model from the multivariable logistic regression together with odds ratios (OR) and 95% confidence intervals (CI)

Variable	Beta Coefficient	OR^a^	95% CI	*p*-value
Intercept	−5.697	–	–	
Categorical Variables				
Male gender	−0.123	0.88	(0.82–0.95)	0.001
Diagnoses in hospital care				
C78 Secondary malignant neoplasm of respiratory and digestive organs	1.009	2.74	(1.39–5.49)	0.004
E11 Type 2 diabetes mellitus	0.317	1.37	(1.13–1.66)	0.001
G40 Epilepsy	0.840	2.32	(1.36–3.95)	0.002
Z93 Artificial opening status	0.791	2.20	(1.22–4.01)	0.009
Diagnoses in open-clinic visits				
A09 Other gastroenteritis and colitis of infectious and unspecified origin	0.559	1.75	(1.09–2.75)	0.02
C79 Secondary malignant neoplasm of other and unspecified sites	0.824	2.28	(1.51–3.41)	< 0.001
C83 Non-follicular lymphoma	0.986	2.68	(1.33–5.37)	0.005
D50 Iron deficiency anemia	0.335	1.40	(1.08–1.80)	0.01
E14 Unspecified diabetes mellitus	0.160	1.17	(1.03–1.34)	0.02
F10 Mental and behavioural disorder due to use of alcohol	0.917	2.50	(1.52–4.09)	< 0.001
G20 Parkinson’s diseasae	0.548	1.73	(1.25–2.38)	< 0.001
I20 Angina pectoris	0.221	1.25	(1.04–1.49)	0.01
I25 Chronic ischaemic heart disease	0.128	1.14	(1.01–1.27)	0.03
I48 Atrial fibrillation and flutter	0.183	1.20	(1.08–1.34)	< 0.001
I50 Heart failure	0.276	1.32	(1.15–1.51)	< 0.001
I73 Other peripheral vascular disease	0.366	1.44	(1.08–1.90)	0.01
J44 Chronic obstructive pulmonary disease	0.520	1.68	(1.44–1.97)	< 0.001
J84 Other interstitial pulmonary disease	0.642	1.90	(1.11–3.20)	0.02
K50 Crohn disease	1.013	2.75	(1.41–5.29)	0.003
K56 Paralytic ileus and intestinal obstruction without hernia	0.727	2.07	(1.13–3.77)	0.02
M05 Rheumatoid arthritis	0.501	1.65	(1.17–2.31)	0.004
N08 Glomerular disorders	1.176	3.24	(1.26–8.51)	0.01
N18 Chronic kidney disease	0.422	1.53	(1.20–1.93)	< 0.001
R07 Pain in throat and chest	0.213	1.24	(1.05–1.46)	0.01
R10 Abdominal and pelvic pain	0.234	1.26	(1.07–1.48)	0.005
R41 Symptoms and signs involving cognitive function	0.359	1.43	(1.14–1.79)	0.002
R42 Dizziness and giddiness	0.245	1.28	(1.10–1.49)	0.002
R55 Syncope and collapse	0.384	1.47	(1.11–1.93)	0.006
R60 Oedema	0.376	1.46	(1.22–1.73)	< 0.001
S00 Superficial injury of head	0.476	1.61	(1.19–2.18)	0.002
S30 Superficial injury of abdomen, lower back and pelvis	0.709	2.03	(1.17–3.53)	0.01
X50 Overexertion and strenuous or repetitive movements	0.780	2.18	(1.16–4.04)	0.01
Continuous Variables				
Age	0.047	1.05	(1.04–1.05)	< 0.001
Non-physician visits	0.009	1.01	(1.01–1.01)	< 0.001
Physician visits	0.019	1.02	(1.01–1.03)	< 0.001
Previous in-ward hospital stays	0.099	1.10	(1.05–1.16)	< 0.001
Emergency room (ER) visits	0.123	1.13	(1.08–1.18)	< 0.001

*OR* Odds Ratio, *CI* Confidence interval

Based on a training sample (*n* = 20,364)

### Outcome using different proportions of predicted cases and different time periods

The outcome of the case-finding model varies depending on the risk score used, with low-risk scores (cut-off value) including a large sample and high-risk scores resulting in a more targeted sample. The choice of risk score level is important in clinical practice since it will affect the proportion of predicted cases (Table 4). It is apparent that an increase in the cut-off value rapidly decreases the number of predicted cases and results in a corresponding loss of sensitivity. An important perspective from a clinical point of view is to decide on a manageable proportion of the predicted population that still enables a clinically meaningful sensitivity. As shown in Table 4, predicted proportions of 40 or 45% result in sensitivities of 62 and 66%, respectively. Using a 40% predicted population, we then investigated how different outcome periods would affect the quality of the predictions.

**Table 4:** Falling proportions of predicted cases and corresponding cut-off values on a validation data set (*n* = 20,364)

Proportion predicted	Cut-off values	No. of true positive cases	No. of false positive cases	No. of true negative cases	No. of false negative cases	Sensitivity	Specificity	Positive predictive value	Negative predicted value
95%	0.101	3960	15,438	889	77	98%	5%	20%	92%
90%	0.108	3869	14,416	1911	168	96%	12%	21%	92%
85%	0.114	3780	13,485	2842	257	94%	17%	22%	92%
80%	0.120	3660	12,651	3676	377	91%	23%	22%	91%
75%	0.127	3544	11,659	4668	493	88%	29%	23%	90%
70%	0.133	3447	10,801	5526	590	85%	34%	24%	90%
65%	0.140	3322	9900	6427	715	82%	39%	25%	90%
60%	0.148	3160	9016	7311	877	78%	45%	26%	89%
55%	0.157	3001	8099	8228	1036	74%	50%	27%	89%
50%	0.165	2862	7303	9024	1175	71%	55%	28%	88%
45%	0.175	2682	6446	9881	1355	66%	61%	29%	88%
40%	0.186	2501	5639	10,688	1536	62%	65%	31%	87%
35%	0.199	2310	4813	11,514	1727	57%	71%	32%	87%
30%	0.215	2050	4004	12,323	1987	51%	75%	34%	86%
25%	0.234	1841	3213	13,114	2196	46%	80%	36%	86%
20%	0.258	1565	2486	13,841	2472	39%	85%	39%	85%
15%	0.294	1257	1775	14,552	2780	31%	89%	41%	84%
10%	0.349	904	1130	15,197	3133	22%	93%	44%	83%
5%	0.446	503	511	15,816	3534	12%	97%	50%	82%

### Sensitivity analysis

The main prediction model was based on unplanned hospital admissions (*n* = 8167), but a model including both planned and unplanned hospital admission (*n* = 9354) resulted in an AUC of 0.68 (95% CI: 0.67–0.69). The variables in the two models were almost identical and 85% of the variables in the planned/unplanned model was included in the unplanned model. Also, a model based on unplanned hospital admission excluding 2166 people who died within the 12 months follow up period was created resulted in an AUC of 0.67 (95% CI: 0.66–0.68). Excluding people resulted in a lower AUC but the model was similar to the main prediction model and 80% of the variables was present in the main prediction model. Two different time intervals were created based on unplanned hospital admission, where 3- (*n* = 2503) and 6-month (*n* = 4664) follow-up models resulted in AUC of 0.70 (95% CI: 0.68–0.71), and 0.69 (95% CI: 0.68–0.70), respectively. Using the lasso method did not improve the AUC (0.69 (95% CI: 0.68–0.70)) compared with the stepwise procedure method.

### External validation

The main prediction model was also tested on two external samples for unplanned hospital admission over the 12 following months. Using the same time period as above for data collection (2015/2016), but for the age group 65–74 (*n* = 51,104) the AUC was 0.68 (95% CI: 0.67–0.69). Using the age group 75 years and older, but for another time point (2012) (*n* = 38,121), the AUC was also 0.68 (95% CI: 0.67–0.69).

## Discussion

We used administrative routine healthcare data in order to develop a prediction model for unplanned admissions of older persons to hospital. Emergency-room visits, age, number of non-physician visits and number of physician visits were the most important variables for the model. The addition of the other 33 variables only slightly increased the AUC. The different sensitivity analyses showed similar AUC. The absence of larger impact by different medical diagnoses on the accuracy of the model, can be explained by the fact that the use of the healthcare system is the ultimate consequence of all diagnoses.

### Strengths and limitations

The main strength of this study in comparison to earlier smaller and more selected studies is the large population including all inhabitants 75 years or older in a county without selection factors like insurance system or specific care providers [19, 20]. The validity of a prediction tool is crucial for its possible usefulness in a broader clinical context [22] e.g. in other countries with similar structures for administrative healthcare data. It may be a weakness of the study that we were unable to include data from other counties or countries. But the external validity of our model was corroborated in two external samples, one using a different time period and one using a younger age group. Another limitation of the model is the lack of socio-economic and socio-demographic data, data not available in the administrative health care data. But considering that the important variables of the model as well its accuracy are strikingly corresponding to a study in an American context supports the validity of the model [19]. There are other risk adjustment-measures for hospitalization, but the AUC values are in the same range as reported in our study [18]. Since the outcome (accuracy) of our model is also in the same range as (or better than) studies in other countries and using similar, but not identical, settings, we modestly assume our data to be generalizable [24].

### Use of the model in a clinical context

High accuracy (expressed as c-statistics) is to be expected for diagnostic tests like medical imaging or polygraph lie detection, but in mores complex settings, like some types of weather forecasting, c-statistics may in fact turn out to be 0.6–0.7 [23]. In a complex system with healthcare of “frail elderly” or “older persons with multi-morbidity” prediction of hospitalization of a population without a clear clinical definition (it is unlikely to obtain accuracy measures much higher than that. The accuracy expectations in a complex clinical context must be reasonable, in order to use the predictive tool in a clinically meaningful way. In a clinical context, sensitivity and specificity must be balanced so that a clinically meaningful outcome of the prediction is obtained. When an intervention is planned, the model must be able to find a reasonable number of the true cases (i.e. $\frac{2}{3}$ or $\frac{3}{4}$). But this cannot be combined with selecting too many false positive cases (low specificity). The model selected in our study, with AUC 0.69, can be regarded as a statistically accurate model which works for a clinically complex population. As illustrated in Table 4, the model must be managed in a clinically relevant context where there is a balance between the number of cases and non-cases selected by the model. We found that a predicted proportion of 40 or 45% of the population is a clinically meaningful reduction of the population to less than half, releasing healthcare resources from the other half with less probable needs. The selected 40 or 45% still contains 62 to 66% of the cases of the whole population. This is a significant enhancement of the probability of reaching the correct target group with a planned proactive intervention. Translated into the reality of a general practitioner (GP) with 2000 listed patients (all ages), he or she would get a list of 50–70 predicted cases. This number of patients that can be screened through and prioritized (from high to low) by the GP who can exclude individuals who are apparently falsely predicted. It should be noted that the positive predicted value for the same proportion of predicted individuals (40%) was 31%. In clinical practice, this is of greater importance than the AUC value itself. If the clinician experiences that 20–30% of predicted individuals are true cases and more than 60% of all cases are detected, our experience is that they find the model to be clinically relevant.

### Prediction enables proactive intervention

The meaning of the prediction was to use it in a clinical setting which during the next implementation phase was for clinical (intervention) purposes [21]. In clinical practice, the predicted population was transferred as patient lists to each primary care centre, who could plan and implement proactive interventions (e.g. home visits, telephone support, GP visits). Such interventions given to a poorly defined group of elderly people in a certain age-range or to a “multi-morbidity-group” with low predictive value for hospitalization are likely to direct healthcare resources towards groups that are not in need of them [21]. And interventions for small, specific groups that can be selected manually (newly hospitalized, specific medical diagnosis like heart insufficiency, “above a certain frailty index score”) will miss large groups of elderly in need of healthcare or largely miss the wider care-flows of geriatric hospital care (low sensitivity), see e.g. [13]. Therefore, our healthcare providers now have decided that prediction of risk (for hospitalization) patients in the 75+ population will be introduced into routine primary care where stratified risk-lists will be used for the planning of proactive team-based intervention.

### Frailty measures or administrative data?

Using clinical instruments with “frailty” as a predictor for hospital care has practical limitations since it requires a face-to-face meeting and also has poor accuracy for prediction of admission to hospital (AUC 0.52–0.57) [13]. In contrast, predictive models based on administrative healthcare data seem more reliable for the prediction of hospital admissions [18, 19, 25]. In clinical practice, using a digital predictive model combined with a geriatric assessment including a frailty measure is likely to be more useful than either instrument alone [21].

## Conclusion

There is strong evidence for the value of geriatric-dedicated assessment, both in hospital and primary care [14, 26–28]. Prediction of the target population for these assessments/interventions enables the healthcare provider to direct proactive resources towards a group in greater need which may increase the capacity and cost-effectiveness of the interventions. We provide a clinically useful prediction model with acceptable accuracy for hospital admissions of older possibly frail persons. We indicate how it can be used in a clinical primary care context and how the healthcare can focus its resources to clinically relevant sub-populations. The method and models used can be generalized and implemented in most healthcare systems with electronic healthcare statistics. Prediction of patients at risk for hospitalization may certainly be one important way for the future healthcare system to meet increasing needs for care, but it must be used sensibly in clinical practice.

## Data Availability

Due to ethical restrictions we are not allowed to submit our data-file outside our research environment. If other scientists want to explore or validate their own models on our data-set we will certainly be of assistance in doing so upon reasonable request to the corresponding author.

## Abbreviations

AUC: Area under the receiver operating characteristics curve
CI: Confidence interval
C-statistics: Concordance-statistics, goodness of fit for binary outcome in a logistic regression
ER: emergency room
glmnet: Lasso and Elastic-Net Regularized Generalized Linear Models
GP: general practitioner
ICD10: International classification of diseases 10th revision
LR: Logistic regression
MASS: Modern Applied Statistics with S
OR: Odds ratio
PPV: Positive predictive value
pROC: Display and Analyze ROC Curves
Ref: Reference value
rms: Regression Modeling Strategies
ROC: Receiver operating characteristics
TRIPOD: Transparent reporting of multivariable prediction model for individual prognosis or diagnosis
